# Characterization of vascular endothelial progenitor cells from chicken bone marrow

**DOI:** 10.1186/1746-6148-8-54

**Published:** 2012-05-14

**Authors:** Chunyu Bai, Lingling Hou, Minghai Zhang, Yabin Pu, Weijun Guan, Yuehui Ma

**Affiliations:** 1 Institute of Animal Sciences, Chinese Academy of Agricultural Sciences, Beijing, 100193, China; 2 College of Life Sciences and Bioengineering, Beijing Jiaotong University, Beijing, 100044, China; 3 College of Wildlife Resources, Northeast Forestry University, Harbin, 150040, China

**Keywords:** Chicken, Biological characteristics, Endothelial progenitor cells, Isolation

## Abstract

**Background:**

Endothelial progenitor cells (EPC) are a type of stem cell used in the treatment of atherosclerosis, vascular injury and regeneration. At present, most of the EPCs studied are from human and mouse, whereas the study of poultry-derived EPCs has rarely been reported. In the present study, chicken bone marrow-derived EPCs were isolated and studied at the cellular level using immunofluorescence and RT-PCR.

**Results:**

We found that the majority of chicken EPCs were spindle shaped. The growth-curves of chicken EPCs at passages (P) 1, -5 and -9 were typically “S”-shaped. The viability of chicken EPCs, before and after cryopreservation was 92.2% and 81.1%, respectively. Thus, cryopreservation had no obvious effects on the viability of chicken EPCs. Dil-ac-LDL and FITC-UAE-1 uptake assays and immunofluorescent detection of the cell surface markers CD34, CD133, VEGFR-2 confirmed that the cells obtained *in vitro* were EPCs. Observation of endothelial-specific Weibel-Palade bodies using transmission electron microscopy further confirmed that the cells were of endothelial lineage. In addition, chicken EPCs differentiated into endothelial cells and smooth muscle cells upon induction with VEGF and PDGF-BB, respectively, suggesting that the chicken EPCs retained multipotency *in vitro*.

**Conclusions:**

These results suggest that chicken EPCs not only have strong self-renewal capacity, but also the potential to differentiate into endothelial and smooth muscle cells. This research provides theoretical basis and experimental evidence for potential therapeutic application of endothelial progenitor cells in the treatment of atherosclerosis, vascular injury and diabetic complications.

## Background

Endothelial progenitor cells (EPCs) are precursors of vascular endothelial cells. Progenitor cells [1]. EPCs originate from bone marrow with similar angioblast and umbilical vein endothelial cells, which together belong to a subgroup of hematopoietic stem cells [2].

There are two types of EPCs that can be detected *in vitro*, early and late EPCs. While early EPCs display a linear growth structure termed spindle-shaped, late EPCs form cobblestone-like, oval shaped strutures [3]. EPCs not only take part in vascularization during embryonic development, but also participate in postnatal vascularization and reparative processes post-trauma [4]. Therefore, EPCs hold extensive prospects for vascular tissue engineering and possible clinical application in coronary artery disease and wound healing [5]. However, the therapeutic use of EPCs remains controversial with many scholars believing that transplantations are reckless. In contrast, there have been cases where clinical transplantation of EPCs for ischemic disease has been successful [6,7]. Therefore, the therapeutic application of EPCs has become more and more attractive.

In addition, there is a close link between diabetic complications and decreased number and activity level of EPCs in vessels [8]. It is hypothesized that the loss of function and reduced number of EPCs leads to cardiovascular complications including induced atherosclerosis, heart disease or apoplexy, which are primary causes of death in diabetic patients [9]. The initial loss of EPC function is believed to relate to increased patient weight [10]. In contrast, the diabetes-related retinopathy is caused by high levels of EPCs in the endocapillaries which promote neurotrophic factor [11]. Thus, EPCs play a key role in diabetes. Moreover, EPCs likely contribute to angiogenesis-associated cancer diffusion [12]. The number of EPCs in circulation is raised after chemotherapy as revealed by Roodhart et al, therefore EPCs could be a novel target for anti-cancer therapy [13]. In summary, EPCs hold great prospects for future medical application, however, more in-depth studies are required to investigate the feasibility of using EPCs in the treatment of the aforementioned diseases. To date, the vast majority of experimental EPCs have been obtained from humans, mouse and other mammals, but rarely from avian species. As a different model species, the chicken has an abundance of EPCs. Thus, in the present study, chicken EPCs were evaluated. EPCs were isolated from the bone marrow of one day old chickens and cultured *in vitro.* Cultured cells were verified to be EPCs by testing for stemness hallmarks: self-renewal capacity and differentiation abilities; and confirming the expression of EPC specific surface markers. This research provides novel insights for the isolation and *in vitro* culture of chicken EPCs, and their possible use for tissue reconstruction in avian species.

## Materials and methods

### Experimental animal

The Institutional Animal Care and Use Committee of Chinese Academy of Agricultural Sciences approved all animal procedure.

Sixty one-day-old Beijing Fatty chickens (*Gallus gallus*) were provided by the Poultry Experimental Base Institute of Animal Sciences, Chinese Academy of Agricultural Sciences, Beijing. All chickens were treated in accordance with NIH and USDA guidelines for the use of animals in research and all experimental procedures involving chickens were conducted in accordance with the protocols and guidelines for agricultural animal research codified by the Committee for Ethics of Beijing, China.

### Experimental reagents

DMEM/F12 (Gibco, USA), special grade fetal bovine serum (Biochrom,Germany) Percoll lymphocyte separating (Pharmasha), Trypsin 1: 250 (Amresco), VEGF, bFGF, IGF-1, PDGF-BB (Invitrogen, USA), rabbit anti chicken CD34, VEGFR-2 polyclonal antibody (MBL, Japan), mouse anti CD133 polyclonal antibody, EDTA, FITC conjugated goat anti mouse secondary antibody IgG, FITC conjugated goat anti rabbit secondary antibody IgG (Zhongshan Golden Bridge, China), mouse antiα-MSA polyclonal antibody (BOSTER),FITC-UEA-1(Sigma, USA), Dil-ac-LDL(Invitrogen, USA).

## Methods

### Isolation of chicken EPCs

Thighs obtained from sacrificed experimental chickens were soaked in 75% alcohol for 3 min. Next, muscles and connective tissues were removed under sterile conditions to obtain the tibias and the metaphyseal was resected to expose the bone marrow cavity. Using a syringe, bone marrow was removed and resuspended in 10 ml serum-free L-DMEM medium containing 100 IU/ml penicillin and 100 μg/ml streptomycin. The bone marrow cell suspension was then carefully layered over 3 ml of 1.077 g/ml Percoll solution in a 10 ml centrifuge tube. The mixture was centrifuged at 400 × g for 20 min at room temperature and the white nebulous layer was collected, washed twice using L-DMEM and centrifuged for a further 5 min at 200 × g. After counting, cells were plated into flasks at 1 × 10^5^ cells/ml, and cultured at 37°C, 5% CO_2._ Half of the medium was changed after three days, and then once every three days thereafter.

### The influences of matrix and medium on EPCs culture

The original passage (P0) EPCs were plated at a density of 1 × 10^5^ cells/well onto 24-well plates coated with either 0.1% gelatin or fibronectin, respectively, then cultured in either DMEM/F12 supplemented with 10% FBS, 10ng/ml VEGF, 10ng/ml bFGF and 10ng/ml IGF-1, dubbed Media A, or M199 supplemented with 10% FBS, 10ng/ml VEGF, 10ng/ml bFGF and 10ng/ml IGF-1, dubbed Media B. The growth dynamics of EPCs were then investigated under the different culture conditions (n = 6/group).

### Estimation of cell viability

EPC viability before and after cryopreservation was detected using the Trypan blue exclusion test as previously described [14]. The number of non-viable cells was determined from a total of 10^4^ counted cells.

### Growth dynamics

To assess growth dynamics, six individual EPCs of passages 1, 5, and 9 were seeded in triplicate in 24-well plates at a density of 1 × 10^5^ cells/well and cultured for seven days. Cells were counted every day thereafter for up to eighth days. The mean cell counts at each time point were used to plot growth curves. Based on this the population doubling time (PDT) was calculated.as follows: PDT = (t-t_0_) lg2/ (lgNt-lgN_0_), where t_0_ = starting time of culture; t = termination time of culture; N_0_ = initial cell number of culture; N_t_ = ultimate cell number of culture.

### Dil-ac-LDL and FITC- UEA-l uptake by EPCs

EPCs of passages 1, 5 and 9 were washed thrice (5 min each) using PBS, then incubated in media containing 12μg/mL Dil-ac-LDL for 4 h at 37°C, 5% CO_2_. Cells were then washed thrice more (5 min each), fixed with 2% paraformaldehyde, and incubated for 1 h with FITC-UEA-1 (10 μg/ml) at room temperature. Cells were observed using the Nikon TE-2000-E confocal microscope.

### Detection of EPC markers

EPCs of passages 1, 5 and 9 were fixed in 4% (m/v) paraformaldehyde for 15 min then washed thrice with PBS (5 min each). Cells were permeabilized using 0.2% (v/v) Triton X-100 for 20 min and washed a further three times (5 min each) with PBS. The cells were blocked in 10% (v/v) goat serum for 30 min, and subsequently incubated in 3% (w/v) bovine serum albumin (BSA) containing the following antibodies: (1) Mouse α Chicken CD133 (1:100); (2) Rabbit α Chicken CD34 (1:500); and (3) Rabbit α Chicken KDR (1:500) for 1 h at room temperature. Next, cells were washed thrice (10 min each) with PBS, and incubated in PBS containing the appropriate fluorescein isothiocyanate (FITC)-conjugated secondary antibody for 1 hr at 37°C. After incubation, cells were washed thrice with PBS (10 min each). The cells were observed using the Nikon TE-2000-E confocal microscope. Ten non-overlapping visual fields were randomly selected. Images were acquired and used to calculate positive ratios.

### Detection of weibel-palade bodies

Weible-Palade bodies were observed by transmission electron microscopy after fixation, dehydration, permeabilization and sectioning of EPCs collected at passages 1, -5 and -9.

### Endothelial differentiation

EPCs were divided into two groups: induced and control. When EPCs attained 60%–70% confluence, cells of the induced group were incubated with endotheliocyte media containing 30ng/ml VEGF, while control cells remained in complete medium alone. Media were refreshed every three days and three weeks later mRNA expression of CD31, CD34 and CD133 was assessed via RT-PCR. Non-induced EPCs were used as a negative control.

### Smooth muscle cell differentiation

The cells were divided into two groups, induced and control. When EPCs attained 60%–70% confluence, cells of the induced group were incubated in endotheliocyte media containing 20ng/ml PDGF-BB, while control cells remained in complete medium alone. The media were refreshed every three days. Two weeks later, mRNA expression of α-MSA and CD133 were assessed via RT-PCR. Non-induced EPCs were used as a negative control.

### Reverse transcription PCR (RT-PCR)

Collect the cells, extract total RNA with Trizol (Invitrogen). Total RNA was reverse transcribed followed by 30 PCR cycles using RNA PCR kit ver 3.0 (TARAKA, China). Information of gene specific primer pairs was listed in Table 1. PCR was performed in a 25μl mixture containing 2.5μl 10 × PCR Buffer (TARAKA, China), 2μl dNTP Mix (2.5mM) (TARAKA, China), 16.75μl ddH_2_O, 0.25μl Ex-Taq (TARAKA, China), 1μl forward and reverse primers, and 1.5μl template cDNA. The cycling conditions consisted of initial 5 min at 94°C 0ne cycle and than followed by 30 cycles of 30s at 94°C (for denaturation), 30s at 50-60°C(for annealing), 2 min at 72°C( for extension). PCR products were detected by 2.5% agarose gel electrophoresis.

**Table 1:** The primer sequences used for RT-PCR

Name	Sequence	Length (bp)	Tm°C
CD34	F:5′GTGCCACAACATCAAAGACG 3′ R: 5′GGAGCACATCCGTAGCAGGA 3′	239	60
VEGFR-2	F: 5′ GGTCGCATGAACATGAAGAA3′ R: 5′ TTGGTAGGGTTTGTAAGGAC3′	248	58
α-MSA	F: 5′AGCACCACTGAATCCCAAAG3′ R: 5′AGTCCAGGGCCACATAACAC3′	347	58
GAPDH	F:5′ TAAAGGCGAGATGGTGAAAG 3′ R: 5′ ACGCTCCTGGAAGATAGTGAT 3′	244	60

### Statistical analysis

Statistical analyses of the data were performed with a one-way ANOVA followed by the Tukey-Kramer honestly significant difference (HSD) test for the three sets of results. A *P*-value of less than 0.05 was considered significant. Statistical analyses were done with a JMP® Statistical Discovery Software (SAS Institute, Cary, NC).

## Results

### Optimization of in vitro culture condition

**Figure 1:**
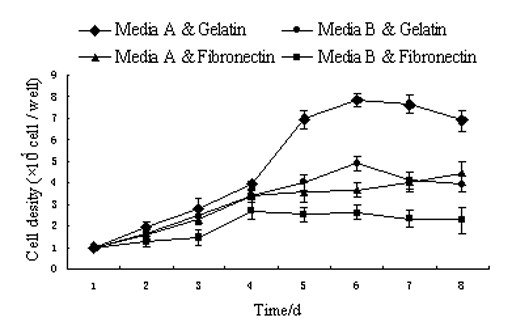
**The growth curves of EPCs under different culture conditions.** The growth curves of EPCs appeared sigmoidal under the “Media A & Gelatin” culture conditions. Other curves did not appear sigmoidal. The population doubling times (PDT) were approximately 31.9 h (Media A & Gelatin), 40.5 h (Media B & Gelatin), 43.6 h (Media A & Fibronectin) and 52.7 h (Media B & Fibronectin). “Media A & Gelatin” was determined to provide the optimal conditions for the proliferation of EPCs *in vitro.*

To determine the optimal culture conditions for chicken EPCs, two adhesive compounds were tested: gelatin (0.1%) and fibronectin. Two types of media, dubbed A and B, were also evaluated. Passage 0 (P0) EPCs (1 × 10^5^) were seeded onto culture plates coated with 0.1% gelatin or fibronectin, and cultured in either media A or media B. EPC growth was monitored by daily counting to generate growth curves. The growth curves suggested that combining media A with 0.1% gelatin coated plates provided the optimal culture system for EPCs (Figure 1).

### EPCs morphological

**Figure 2:**
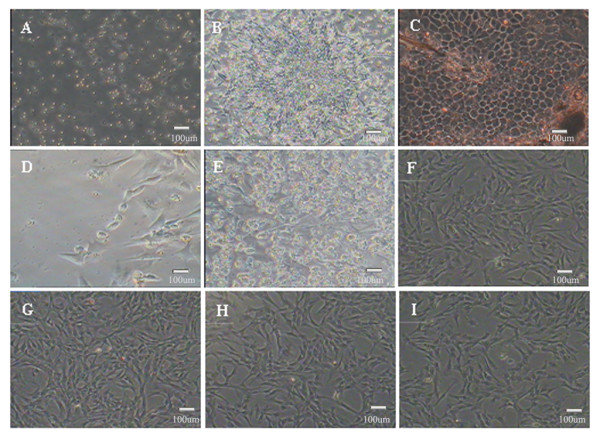
**Morphology of chicken EPCs*in vitro*.A.** Cells plated onto microplates after 0 hours; **B.** ’blood island’ structures of the EPCs; **C.** slabstone-like appearance of the EPCs; **D.** linear growth of the EPCs; **E.** P1 EPCs before passage; **F.** P3 EPCs before passage; **G.** P5 EPCs before passage; **H.** P7 EPCs before passage; **I.** P9 EPCs (bar = 100μm).

To further characterize the chicken EPCs, morphological changes were recorded. The isolated bone marrow mononuclear cells were initially round or irregularly-shaped (Figure 2A), however within 48 h they adhered and began growing. The cells proliferated quickly, displaying obvious karyokinesis and differing morphology including fusiform, triangular and irregular shapes. After 5 days in culture, colonies formed,where rounded cells were found in the middle of clusters, but peripheral cells were more fusiform in shape, and radiated from the centre as single cells. These clusters were termed, “blood islands” (Figure 2B). Linear arrangements of cell structures were also observed (Figure 2C). Approximately eight days after plating, the cells attained 70%–80% confluence and were passaged. The average passage time was 3–5 days, and the cells could be cultured up to P11 at most (Figure 2).

### Cryogenic preservation and resuscitation

Upon reaching P11, most cells displayed features representative of senescence such as blebbing and karyopyknosis. After resuscitation, the cells began to adhere at 24 h and grew rapidly thereafter. There was no significant difference in growth and morphology between cells before and after cryopreservation (Table 2).

**Table 2:** The viability of EPCs before and after cryopreservation was determined using the Trypan blue exclusion test

Category	Endothelial Progenitor Cells(%)
Before Frozen	92.2 ± 0.21
After Reviving	81.1 ± 0.43

### Growth kinetics

Growth and proliferation of EPCs were similar at P1, P5 and P9 according to the growth curves (data not shown). After a latency phase of 1–3 days, cell growth entered the logarithmic phase, and reached the plateau phase at approximately day 7. The population doubling time (PDT) was determined to be 32.5h, 31.9 h and 35.6h for P1, P5 and P9, respectively.

### Dil-ac-LDL and FITC-UEA- detection

**Figure 3:**
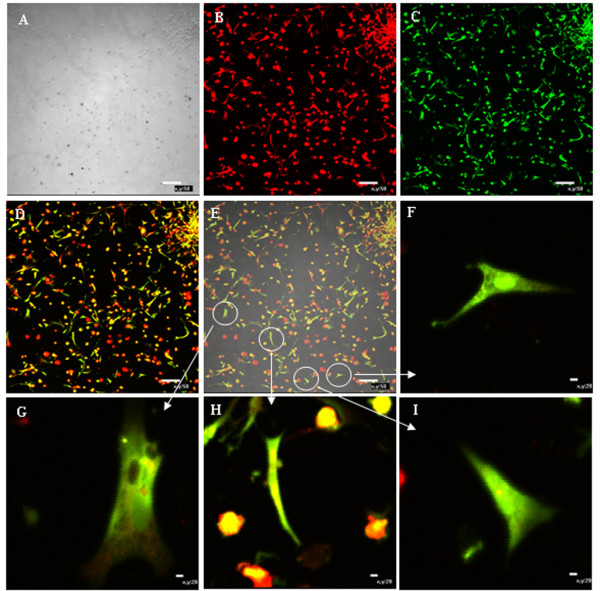
**Chicken EPC uptake of DiI-ac-LDL and FITC-UEA-1.** EPCs were incubated with DiL-ac-LDL and FITC-UEA-1, uptake was then assessed by immunofluorescent microscopy. DiL-acLDL (red); FITC-UEA-1 (green), differentiated EPCs (yellow). (bar = 50μm) **A:** Phase contrast; **B:** Dil-acLDL+; **C:** FITC-UAE-1+; **D:** Dil-acLDL & FITC-UAE1+ C; **E:** Merged; **F, H ,G ,I:** View fields that zoom in from figure E.

EPCs have the ability to uptake Dil acetylated low density lipoprotein (Dil-ac-LDL) and FITC-Oxytropis lectin 1 (FITC-UEA-I). Thus the capacity of chicken EPCs to uptake these two proteins was tested. Both Dil-ac-LDL and FITC-UEA-1 were detected in the chicken EPCs. Dil-ac-LDL (red) was predominantly localized in the cytolymph while FITC-UEA-1 (green) was mostly in the plasma membrane Fluorescent yellow EPCs were also detected when both Dil-ac-LDL and FITC-UEA-1 were absorbed [15],[16] (Figure 3).

### Immunofluorescence

**Figure 4:**
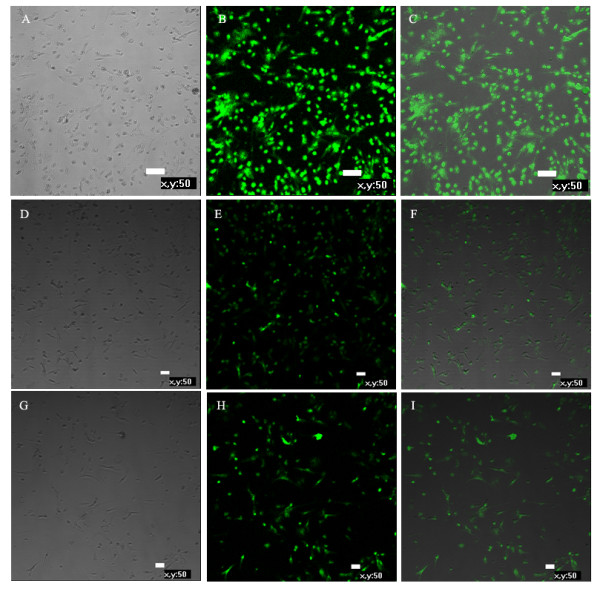
**Expression of EPC surface markers in chicken EPCs.** Expression of the EPC-specific markers CD133, CD34 and VEGFR-2 were examined by immunofluorescent labeling in chicken EPCs. CD133 marks both hematopoietic stem cells and EPCs. Its expression is gradually lost upon differentiation of EPCs into mature cells. **A,D,** and **G**. Phase contrast; **B.** VEGFR-2+; **E.** CD133+; **H.** CD34; **C,F,** and **I.** Merged.

CD133, CD34 and VEGFR-2 are specific markers of EPCs. Thus, to further confirm the identity of chicken EPCs, we examined the expression of these markers in P1, P5 and P9 cells by immunofluorescent staining. We found that the cells expressed the three markers at each passage number (Figure 4).

### The weibel-palade body detection

**Figure 5:**
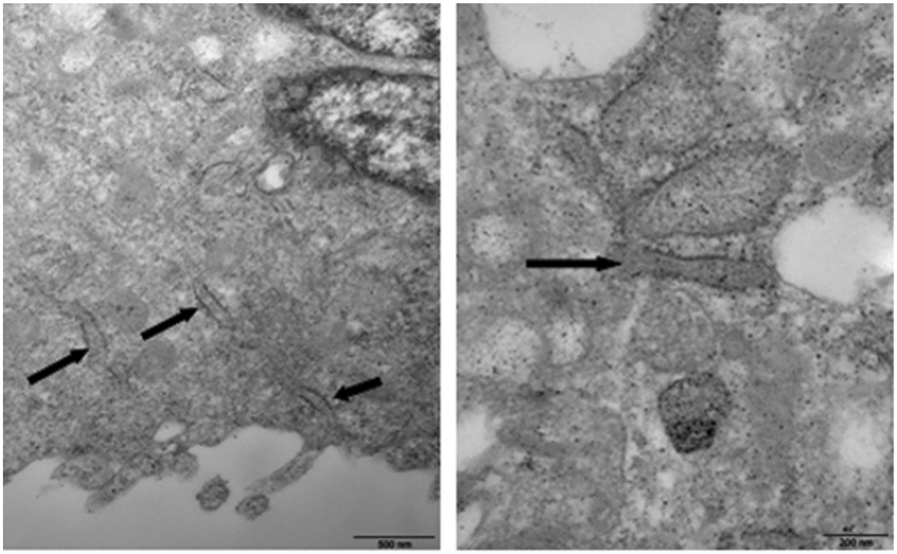
**Weibel-Palade bodies of chicken EPCs under transmission electron microscope.** Weible-Palade bodies, organelles characteristic of endothelial cell lines are indicated. Phagocytic vesicles, mitochondria, and endoplasmic reticulum are also indicated. Their presence suggests active cellular metabolism. (bar = 600μm).

Weible-Palade bodies are endothelial specific organelles. Indeed, under the transmission electron microscope, Weible-Palade bodies were observed in chicken EPCs. In addition, many organelles such as phagocytic vesicles, mitochondria, and endoplasmic reticulum, were also observed, indicative of active cell metabolism in the chicken EPCs (Figure 5).

### Endothelial differentiation of the EPCs

**Figure 6:**
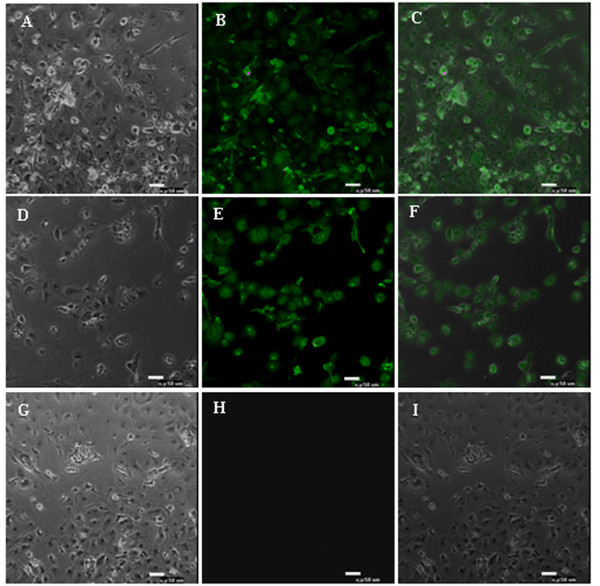
**Identification of endothelial cells by immunofluorescent labeling.** EPC morphology changed from spindle to rounded or irregular in shape, until the cells were very closely located and became cobblestone-shaped. The EPCs were negative for the stem cell marker CD133. **A, D** and **G.** Phase contrast; **B,E** and **H.** Merge; **C.** CD34+; **F.** KDR +; **H.** CD133-.

**Figure 7:**
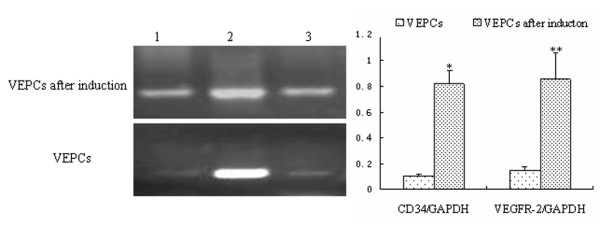
**Detection of EPC markers by RT-PCR.** The expression of CD34 and VEGFR-2, endothelial cell specific markers were significantly different (P < 0.05) before and after induction of differentiation as detected by RT-PCR. Data were analyzed using the Gelpro32 software. 1. CD34; 2. GAPDH; 3. VEGFR-2.

The capacity for chicken EPCS to differentiate into endothelial cells was tested by induction with VEGF and subsequent morphological and phenotypic analysis. Ten days after induction of cells with VEGF, the cell morphology changed from spindle- to round- or irregularly-shaped, until the cells were very closely located to each other and developed a cobblestone pattern (Figure 2D). To confirm that differentiation had occurred, the expression of endothelial markers was examined in the VEGF-induced EPCs by immunofluorescent staining. Induced cells were positive for the endothelial markers CD34 and VEGFR-2, but negative for the progenitor cell marker CD133 (Figure 6). In addition, CD34 and VEGFR-2 mRNA expression before and after differentiation of EPCs was monitored by RT-PCR. Analysis of the data using the software Gelpro32 suggested that induction of EPC differentiation correlated with upregulation of CD34 and VEGFR-2 gene expression (Figure 7). Together, these data indicate that the chicken EPCs can be induced to differentiate into endothelial cells *in vitro* (Figure 7).

### Smooth muscle cell differentiation of the EPCs

**Figure 8:**
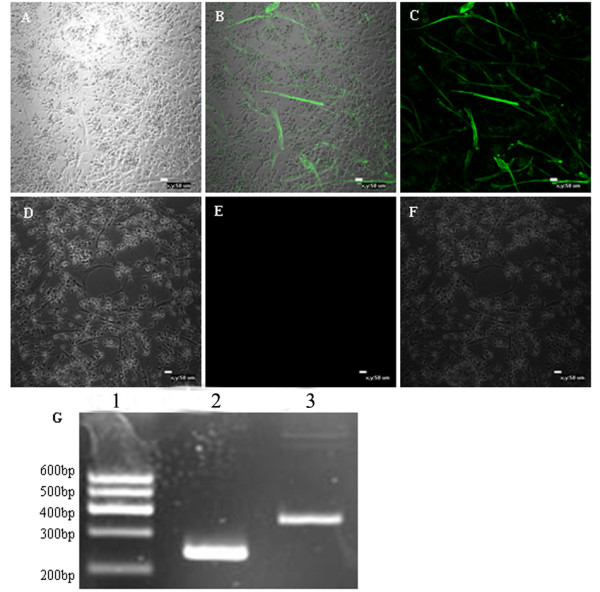
**Smooth muscle cells were identified by immunofluorescence and RT-PCR.** EPC morphology changed after induction with PDGF-BB at day 7, whereby the cells became longer in shape and formed structures typical of muscle cells. The induced cells were negative for the stem cell marker CD133 and positive for the smooth muscle cells marker α-MSA, as detected by immunofluorescence and RT-PCR. **A** and **D**. Phase contrast; **B.** α-MSA+; **E.** CD133- **C.** and **F.** Merge; **G.** smooth muscle cells marker α-MSA detection via RT-PCR, 1: Marker. 2: GAPDH. 3: α-MSA.

The ability of chicken EPCs to differentiate into smooth muscle cells was also tested. The cell morphology was changed at seven days after induction with PDGF-BB. The cells became longer and formed structures typical of muscle cells. The expression of α-MSA, a specific marker for smooth muscle cells, and CD133, a marker for endothelial progenitor cells, was detected by immunofluorescence and RT-PCR. In both assays, PDGF-BB-induced EPCs were α-MSA positive and CD133 negative (Figure 8), suggesting that EPCs can be induced with PDGF-BB to differentiate into smooth muscle cells *in vitro*.

## Discussion

EPCs (also known as CD34^+^CD133^+^VEGFR-2^+^ cell) were first isolated from peripheral blood by Asahara et al in 1997 [1], and have the ability to differentiate into mature endothelial cells and take part in angiogenesis. EPCs and hematopoietic stem cells originate from angioblasts and normally form blood islands [17]. EPCs are usually isolated from umbilical cord blood or peripheral blood, however, approximately 3% of EPCs are found in bone marrow mononuclear cells [18]. In this study, we characterized EPCs isolated from chicken bone marrow.

The isolation methods of EPCs *in vitro* include flow cytometry, MASC (Magnetic Activated Cell Sorting) and density centrifugation. Density centrifugation is usually used for mononuclear cells such as hematopoietic cells and EPCs in blood. Percoll is made of saccharose and characterized by high density, low osmotic pressure and non-toxicity. For the culture conditions, Medium B used in conjunction with fibronectin as a matrix, has previously been reported to provide the best proliferative conditions for mammalian cell types *in vitro*[1][18]. However, it was not the best for our chicken-derived cell cultures. In comparison to other mammal-derived EPCs, the proliferation of chicken EPCs was slower and not easy to rapidly passage. Because DMEM/F12 (Medium A) has greater nutritive constituents than M199 (Medium B), it was used to maintain chicken EPCs in culture. We speculate that the different media preference is related to species-specific characteristics.

The identification criteria of EPCs include cell morphology, specific antigen markers and self-renewal ability. The morphology of EPCs changes from short spindle-shaped cells, cell colonies and linear arrays to a typical cobblestone-like shape during differentiation to mature endothelial cells [19]. CD34^+^CD133^+^VEGFR-2^+^ cells are considered putative EPCs by most researchers [20][21]. CD133 (also known as AC133) is a marker for hematopoietic stem cells and EPCs, and is gradually lost as EPCs differentiate into mature endothelial cells. CD34 is also an antigen marker for hematopoietic and endothelial cell lines. VEGFR-2 (also known as FLK-1 and KDR) is a specific marker for endothelial cells. vWF also functions as a specific marker for endothelial cell lineage. It is produced by endothelial cells and located in the cytoplasm of Weibel-Palade bodies. Thus vWF and Weibel-Palade bodies serve as specific markers for endothelial identification [22][23]. Moreover, the specific ability of endothelial cells to uptake both Dil-ac-LDL and FITC-UEA-1 can be used to identify endothelial cell types. In this experiment, EPCs were identified through detection of the markers CD34, CD133 and VEGFR-2 using immunofluorescence. Uptake of fluorescently labeled Dil-ac-LDL and FITC-UEA-1also revealed that the EPCs were double positive for these indicators. Moreover, Weibel-Palade bodies were observed by transmission electron microscopy. Thus the chicken EPCs retained characteristics typical of EPCs *in vitro*.

EPCs are responsible for the construction of the blood vessel lumen, and are able to differentiate into mature endothelial cells and smooth muscle cells. There are many factors influencing the differentiation of EPCs into mature endothelial cells, among which the central factor is VEGF. Apart from inducing the differentiation of EPCs, VEGF also induces the differentiation of other stem/progenitor cells into mature endothelial cells [19]. The underlying mechanisms are far from clear, except that the VEGF receptor (R)-2 is located on endothelial cell membranes, and that activation of protein kinase B by VEGF initiates various intracellular signaling pathways, which promote the growth and differentiation of cells through the activation of inositol triphosphate [24]. In our study, the concentration of VEGF used to induce differentiation was five-fold higher than that contained within the growth medium, thus greatly promoting differentiation of EPCS into mature endothelial cells. Through RT-PCR, CD34 and VEGFR-2 mRNA expression were found to be significantly different (*P* < 0.05) before and after VEGF-induced differentiation of EPCs into mature endothelial cells. Thus, cultured chicken EPCs can develop into mature endothelial cells in response to VEGF as identified by morphological and phenotypic characteristics.

EPCs are also known to develop into smooth muscle cells. Smooth muscle cells are the main functional cells of the vascular mesosphere and are involved in the synthesis of the vascular matrix and vasomotor function. Platelet-derived growth factor-BB (PDGF-BB) was used to induce EPC differentiation into smooth muscle cells. Seven days post-induction, cells with myoid morphology were observed. In accordance with the morphology, immunolabelling for the stem cell marker CD133 was negative in the inducted cells, while expression of the smooth muscle cell specific marker α-MSA was positive as determined using immunofluorescence and RT-PCR. Together, these data indicate that chicken EPCs can be differentiated into both mature endothelial cells and smooth muscle cells by stimulation with VEGF and PDGF-BB, respectively, *in vitro*.

## Conclusion

In conclusion, this study establishes an optimized method for the isolation and culture of chicken EPCs as suggested by characterization of cell morphology, surface markers and biological features. We also demonstrate that chicken EPCs can be induced to differentiate into endothelial and smooth muscle cells, which supports the notion that the chicken EPCs retained multipotency. This study not only provides a technological platform for the establishment of a chicken EPC bank, but also proposes a new method to preserve the valuable genetic resources of chicken and other poultry.

## Authors’ contributions

CB carried out the cell culture, participated in the cell assay and drafted the manuscript. LH carried out the detection of Weibel-Palade bodies. YP participated in drafted the manuscript. MZ participated in performed the statistical analysis. WG and YM conceived of the study, and participated in its design and coordination. All authors read and approved the final manuscript.
